# Behavioural traits of rainbow trout and brown trout may help explain their differing invasion success and impacts

**DOI:** 10.1038/s41598-022-05484-5

**Published:** 2022-02-02

**Authors:** Ciara L. O. McGlade, James W. E. Dickey, Richard Kennedy, Shannon Donnelly, Clare-Ann Nelson, Jaimie T. A. Dick, Gareth Arnott

**Affiliations:** 1 Institute for Global Food Security, School of Biological Sciences, Queen’s University Belfast, 19 Chlorine Gardens, Belfast, BT9 5DL Northern Ireland UK; 2 Leibniz-Institute of Freshwater Ecology and Inland Fisheries (IGB), 12587 Berlin, Germany; 3 Institute of Biology, Freie Universität Berlin, 14195 Berlin, Germany; 4 AFBI Aquatics Group, River Bush Salmon Station, Church Street, Bushmills, BT57 8QJ Northern Ireland UK

**Keywords:** Behavioural ecology, Freshwater ecology, Invasive species, Animal behaviour

## Abstract

Animal behaviour is increasingly recognised as critical to the prediction of non-native species success and impacts. Rainbow trout and brown trout have been introduced globally, but there appear to be differences in their patterns of invasiveness and ecological impact. Here, we investigated whether diploid rainbow trout and diploid and triploid brown trout differ among several key behavioural measures linked to invasiveness and impact. We assessed activity, boldness, aggression, and feeding, using open field, novel object, shelter, mirror, feeding, and functional response experiments. We also tested within each fish type for behavioural syndromes comprising correlations among activity, boldness and aggression. Rainbow trout were more active and aggressive but less bold than diploid and triploid brown trout. In small groups, however, rainbow trout were bolder than both types of brown trout. Diploid brown trout were more active and bolder than triploids when tested individually, and had a higher functional response than both rainbow trout and triploid brown trout. In terms of behavioural syndromes, there was no association between activity and boldness in rainbow trout, however, there was in both brown trout types. The increased activity and aggression of rainbow trout may reflect an increased stress response to novel situations, with this response reduced in a group. These results suggest that rainbow trout do not manage their energy budgets effectively, and may explain why they have limited survival as invaders. In addition, the lower functional response of rainbow trout may explain why they are implicated in fewer ecological impacts, and the triploidy treatment also appears to lower the potential impact of brown trout. Comparative analyses of multiple behaviours of invasive species and genetic variants may thus be key to understanding and predicting invader success and ecological impacts.

## Introduction

Behaviour is increasingly recognised as an important metric for the explanation of the success or failure of non-native species establishment and spread^1–3^. For example, in several species, e.g. mosquitofish and gobies, individuals at the “invasion front” are bolder, more active, and have faster metabolisms than individuals in more established populations^4–7^, suggesting that these behavioural traits promote success at different invasion stages^8^. At the establishment stage of invasion, increased aggression and greater foraging effort may help to out-compete native analogues^9,10^. Additionally, shyer individuals may suffer less predation than bolder individuals, and reactive (passive but more flexible in behaviour) individuals may respond more effectively to changes in the environment, promoting their survival and thus invasiveness^11^.

Differences in behaviour among native and non-native species can also affect the ecological impact of invaders, that is, changes in native populations of species due to interactions such as predation^12,13^. For example, more aggressive and bolder individuals may have a greater impact on native analogues through direct or indirect interactions, leading to displacement and even extirpation of natives^9,14^. The ecological impact of non-native species on potential prey can also be predicted by comparative functional response experiments, comparing invaders with native trophic analogues^15,16^. Functional responses, which quantify *per capita* feeding rates, can also vary with invasion stage^17^ and have been correlated with certain behaviours. For example, smaller crabs with higher activity levels have a higher functional response, possibly because greater activity levels indicate more time spent actively foraging^18^.

Rainbow trout and brown trout are both economically and ecologically valuable native species and are also among the most impactful invasive species worldwide, with a long history of introductions in many different countries^19–21^. Both species have successfully established invading populations in Australasia^22^, Japan^23^, South Africa^24^, the USA^25^, and elsewhere. The success of rainbow trout in Europe (where brown trout is native) is, however, limited, and in the British Isles, for example, despite many large escapes/releases, there is currently only one known self-sustaining rainbow trout population (in the River Wye, Derbyshire^26^). Where both trout species have been introduced as non-native species, brown trout appears to be a more successful invader with a greater ecological impact on native species. For example, in New Zealand and Australia, brown trout have been implicated in many more negative effects than rainbow trout^22^. In Japan, brown trout has a higher establishment success compared with rainbow trout, despite a seemingly lower introduction intensity of the former^23^. In Colorado, brown trout were found to dominate in areas where both species were introduced, however, a catch and release program benefitted rainbow trout over brown trout, suggesting that rainbow trout may be more vulnerable to angling^27^. In Chilean Patagonia, however, rainbow trout were found to successfully invade a greater area than brown trout^28^, although brown trout were still found to have a greater ecological impact on the native galaxiids.

Different types of behaviour can be correlated with each other in animals, with these correlations termed “behavioural syndromes”^29–31^, such as the correlation between aggression and boldness in sticklebacks^32^, and live-bearing *Poecilia paras* fish exhibit a behavioural syndrome of aggression, boldness and exploration^33^. In invasive species, it has been hypothesized that individuals at the invasion front are likely to exhibit correlations of boldness, aggression and activity, since these traits are associated with dispersal tendency^4,34^.

Most rainbow trout and brown trout which have been released worldwide and are currently released today, however, are not of wild origin, and are instead of highly domesticated strains^21^. The process of domestication can also select for different phenotypes^35^, including suites of correlated behaviours, with increased boldness, greater risks taken when foraging, and increased aggression selected for in intensive aquaculture^36^. While these selected traits can reduce survival in a natural environment, through reducing predator-avoidance behaviours^37,38^, it is also possible that they may lead to greater invasiveness or impact in certain scenarios.

One measure to ostensibly prevent the establishment of invasive trout is a triploidy treatment to prevent reproduction in the wild^39^. However, this raises the question of whether triploid individuals may have a greater or lesser direct ecological impact as a consequence of their different genetics and physiology^40^. Chatterji et al. found that there was little difference in the impact of mixed-sex diploids or all-female triploid stocked brown trout on wild analogues^41^, however triploid brook trout have been found to grow more slowly and are more prone to stress than their diploid counterparts, suggesting that their ability to adapt to the wild and exert impact may be reduced^42^. Triploid Atlantic salmon parr also have a reduced growth rate compared to diploids^43^, however in triploid rainbow trout the opposite was found to be true, suggesting that the influence of the triploidy treatment may not generalize across salmonids^44^. Thus, where possible, ploidy variants of invasive species should be incorporated into behavioural studies.

Although the behaviour of many trout species has been studied previously, there have been very few direct comparisons between rainbow trout and brown trout. Lines of rainbow trout bred for different cortisol responses exhibited different behaviours under stress^45,46^, and in brown trout there is evidence of personality^47^ and a behavioural syndrome comprising activity and aggression^48^. Thus, better understanding the behavioural differences between these two species may help to explain why one is more successful or impactful as an invader in cases where both have been released. The survival of both species from fry to juvenile is a critical period influencing the risk of establishment, as young fish exhaust their yolk sac and must then forage independently^49,50^, with intense competition over foraging territories necessitating aggression^50^. Therefore, in this study we compare several key behavioural traits (activity, boldness and aggression) for both species (including both ploidy variants of brown trout) at the fry stage, as well as measures of potential impact (feeding rate and functional response i.e. *per capita* impacts). We use measures of these traits to assess the evidence for behavioural syndromes in the three types of trout, and interpret our results in the ecological context of the differential success and ecological impacts of these trout as invaders, since all three types are released and often accidentally escape from aquaculture facilities.

We thus carried out a series of previously validated tests on diploid rainbow and both diploid and triploid brown trout. We first assessed boldness and activity using an open field test^51^ a paired novel object^52,53^ and disturbance test (tapping of the novel object on the water surface), and a paired shelter and simulated predation test (since the use of overhanging shelters enables fish to evade predation). A mirror test was used to assess aggression^54,55^ as well as an additional measure of boldness. We then used a fixed density feeding test to assess maximal feeding rate and a comparative functional response experiment to quantify any differences in *per capita* feeding behavior that may explain and predict the generally lower known field ecological impacts of rainbow as compared to brown trout^22,23,27,28^. Finally we used a group test to examine the effect of conspecifics on a boldness measure. We performed linear mixed effects modelling on each of the variables recorded within each test, as well as correlations between the different types of behavior within each trout type to assess the presence of behavioural syndromes. By performing all of these tests, we were able to build a comprehensive picture of the behavioural dynamics and syndromes of rainbow and brown trout, while also expanding our comparison to incorporate the effects of triploidy.

## Results

### Open field test

**Figure 1:**
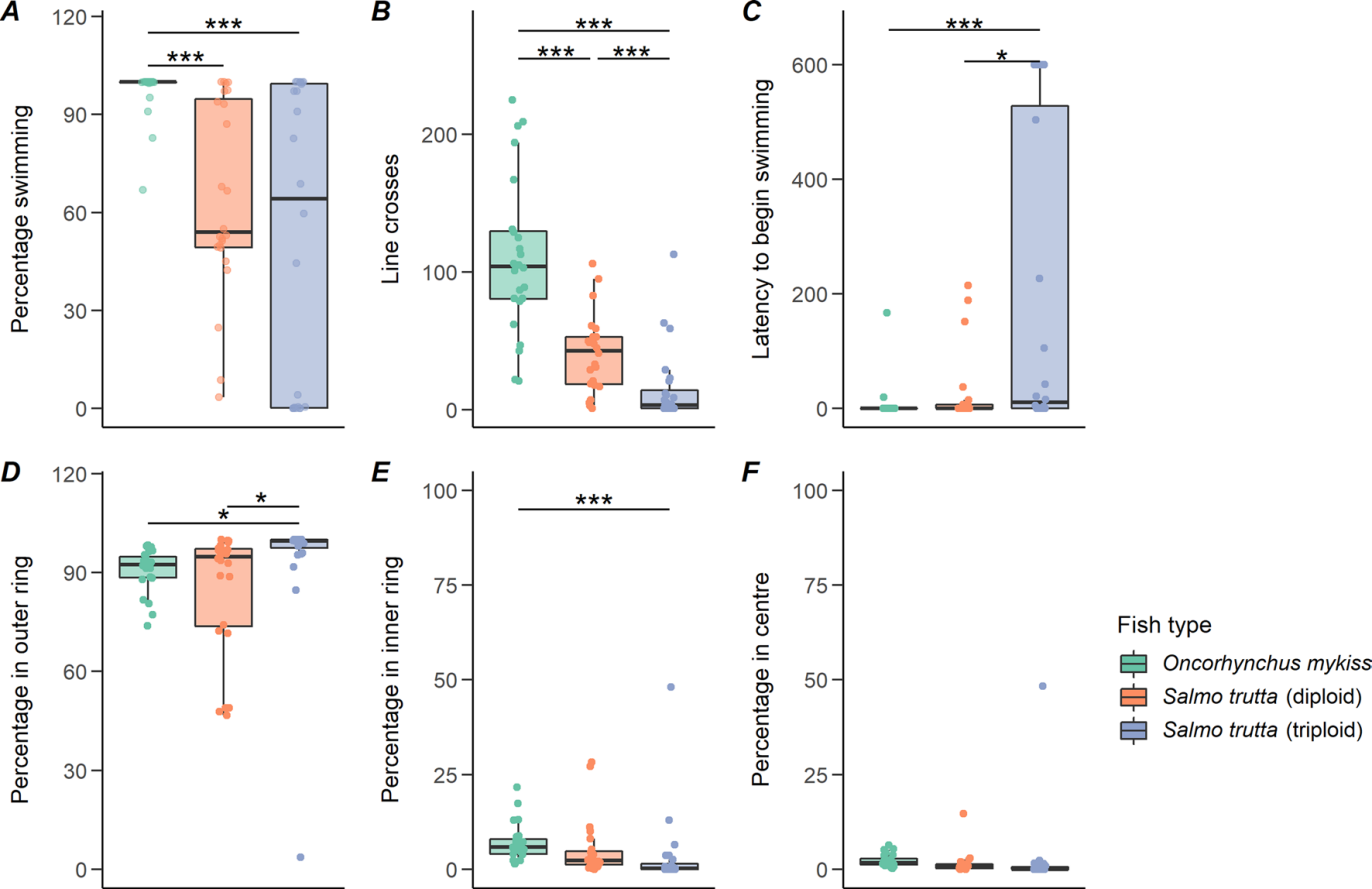
Boxplots showing the variables measured during the open field test, with median and interquartile range plotted and overlayed with raw data points. Asterisks show significance values (* p < 0.05, ** p < 0.01, *** p < 0.001). (**A**) Percentage time spent swimming, (**B**) total number of line crosses, (**C**) the latency to begin swimming from the start of the recording, (**D**) percentage time spent in the outer ring, (**E**) percentage time spent in the inner ring, (**F**) percentage time spent in the centre of the arena.

Rainbow trout spent a greater percentage time swimming than both diploid brown trout (t = 5.92, df = 66, *p* < 0.001) and triploid brown trout (t = 4.98, df = 66, *p* < 0.001), with no difference in percentage time swimming between the two brown trout types (Fig. 1A). Rainbow trout had significantly more line crosses than both diploid (t = 4.64, df = 66, *p* < 0.001) and triploid (t = 8.66, df = 66, *p* < 0.001) brown trout, and diploid brown trout had more line crosses than triploids (t = 4.54, df = 66, *p* < 0.001) (Fig. 1B). There was no difference between rainbow trout and diploid brown trout in latency to begin swimming, but rainbow trout were significantly faster than triploids (t = 4.05, df = 66, *p* < 0.001) as were diploid brown trout (t = 2.39, df = 66, *p* < 0.05) (Fig. 1C).

For the location data, rainbow trout spent significantly less time in the outer ring than triploid brown trout (t = 2.50, df = 66, *p* < 0.05) and diploid brown trout spent less time in the outer ring than triploids (t = 2.46, df = 66, *p* < 0.05). Rainbow trout also spent significantly more time in the inner ring than triploid brown trout (t = 3.83, df = 66, *p* < 0.001) (Fig. 1D–F). All other comparisons were non-significant. There was no significant effect of body mass on any of the behaviour variables measured in the open field test.

These results illustrate that rainbow trout is more active than both brown trout types across two measures within this test, with diploid brown trout more active than triploid brown trout across a single measure. Rainbow trout were also significantly bolder than triploid brown trout across two measures, and diploid brown trout were significantly bolder than triploid brown trout across a single measure.

### Disturbance/novel object test

**Figure 2:**
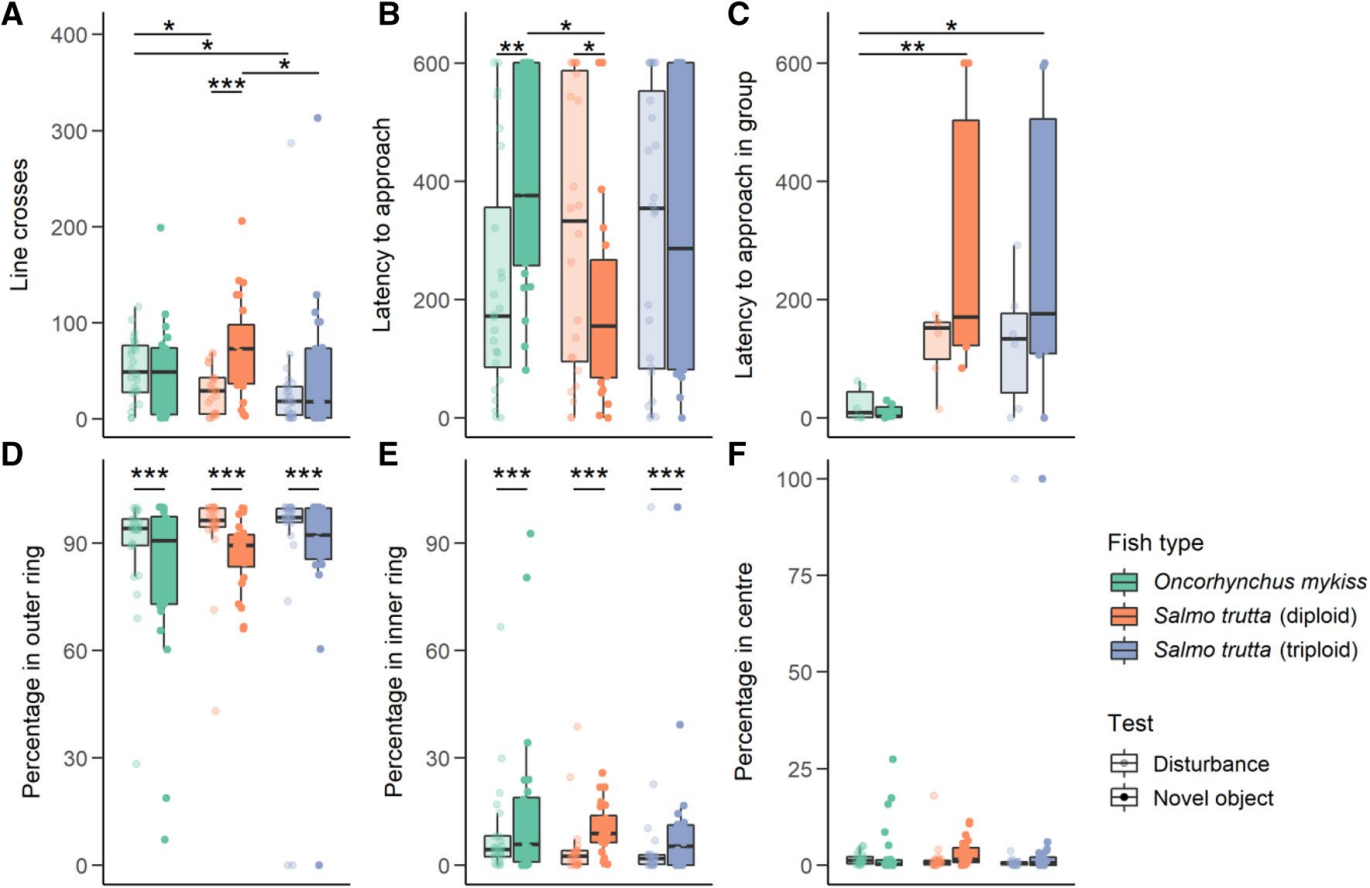
Boxplots showing the variables measured during the disturbance and novel object tests, including the group test, with median and interquartile range plotted and overlayed with raw data points. Asterisks show significance values (* p < 0.05, ** p < 0.01, *** p < 0.001). (**A**) Total number of line crosses, (**B**) the latency to begin swimming from the start of the recording in the individual tests, (**C**) the latency to begin swimming from the start of the recording in the group tests, (**D**) percentage time spent in the outer ring, (**E**) Percentage time spent in the inner ring, (**F**) percentage time spent in the centre of the arena.

In the disturbance test, rainbow trout had significantly more line crosses than diploid brown trout (t = 2.53, df = 64, *p* < 0.05), and triploid brown trout (t = 2.62, df = 64, *p* < 0.05), but there was no significant difference between diploid and triploid brown trout (Fig. 2A). There was also no significant effect of fish type on latency to approach the centre in the disturbance test (Fig. 2B).

In the novel object test, diploid brown trout had more line crosses than triploids (t = 2.8, df = 64, *p* < 0.05), but remaining comparisons across fish type for line crosses were not significant (Fig. 2A). Rainbow trout were significantly slower to approach the novel object than diploid brown trout (t = 2.96, df = 66, *p* < 0.05), but there were no significant comparisons with triploid brown trout (Fig. 2B).

The type of test (disturbance or novel object) affected the three types of fish differently. There was no significant effect of the novel object on the number of line crosses for rainbow trout or triploid brown trout, but diploid brown trout had significantly more line crosses in the presence of the novel object (t = 3.9, df = 69, *p* < 0.001) (Fig. 2A). The order of the test in the series was also significant, with fewer line crosses as the test was conducted later in the day (t = 2.51, df = 64, *p* < 0.05). Rainbow trout were significantly slower to approach the central ring in the novel object than in the disturbance test (t = 2.91, df = 69, *p* < 0.01) whereas diploid brown trout were significantly faster to approach the central ring in the novel object test (t = 2.12, df = 69, *p* < 0.05), with no significant effect in triploids (Fig. 2B). The three types of fish did not differ in the percentage time spent in each part of the arena, but the type of test had a significant effect. Significantly less time was spent in the outer ring (t = 3.64, df = 71, *p* < 0.001) and significantly more time in the inner ring (t = 3.94, df = 71, *p* < 0.001) in the novel object compared to the disturbance test (Fig. 2D–F).

The results of this paired test show rainbow trout to be more active than both brown trout types in the disturbance test and less bold than diploid brown trout in the novel object test. Diploid brown trout were also more active than the triploid trout in the novel object test.

### Shelter/predation test

**Figure 3:**
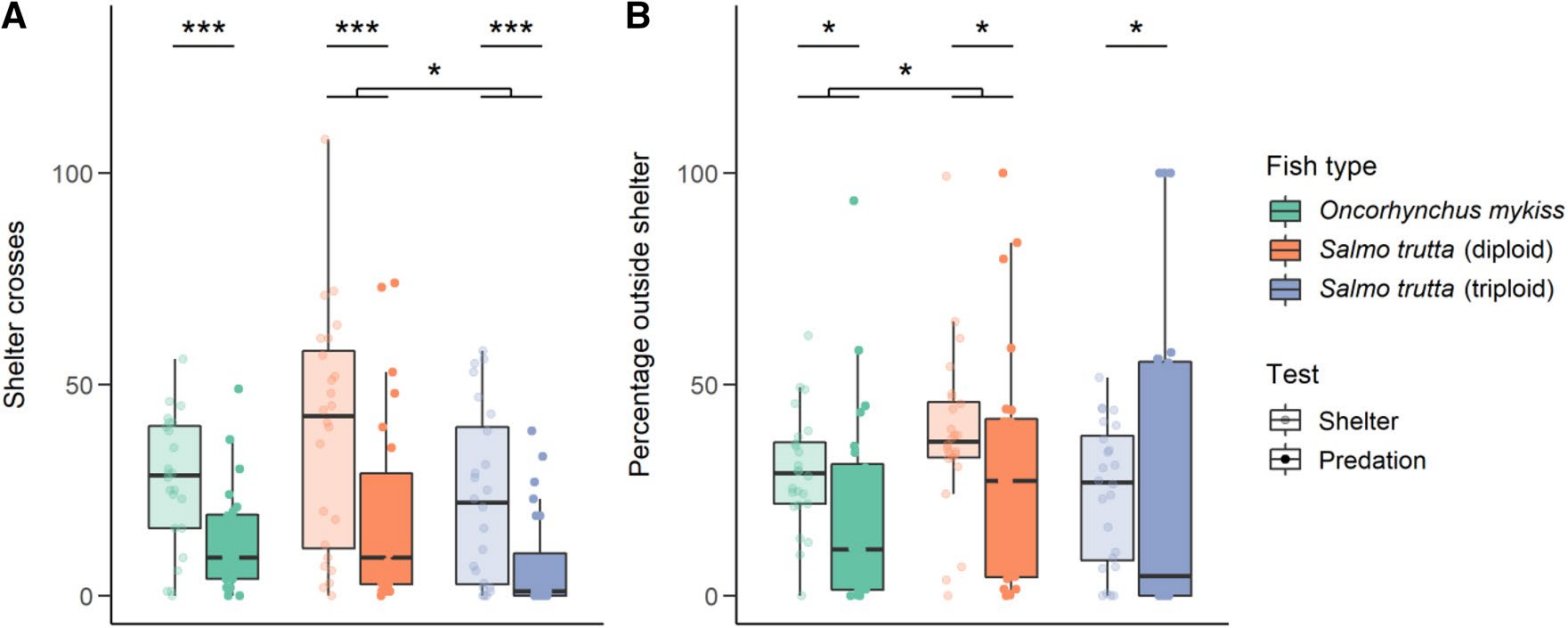
Boxplots showing the variables measured during the shelter and predation tests, with median and interquartile range plotted and overlayed with raw data points. Asterisks show significance values (* p < 0.05, ** p < 0.01, *** p < 0.001). (**A**) Total number of shelter crosses, (**B**) Percentage time spent outside the shelter.

There was no significant difference between rainbow trout and diploid or triploid brown trout for number of shelter crosses, but diploid brown trout had significantly more than triploid brown trout (t = 2.59, df = 66, *p* < 0.05) (Fig. 3A). For both tests, rainbow trout spent significantly less time outside the shelter compared to diploid brown trout (t = 2.04, df = 66, *p* < 0.05), although no comparisons with triploid brown trout were significant (Fig. 3B). There was no significant interaction between type of fish (rainbow trout, diploid or triploid brown trout) and type of test (whether shelter or predation). Instead, number of shelter crosses was significantly decreased by the predation effect (t = 7.30, df = 71, *p* < 0.001) (Fig. 3A), and the percentage time spent outside the shelter also decreased (t = 2.36, df = 71, *p* < 0.05) (Fig. 3B) compared to the shelter test for all three types of fish.

The results of this paired test show rainbow trout to be less bold than diploid brown trout, and diploid brown trout more active than triploid brown trout.

### Mirror test

**Figure 4:**
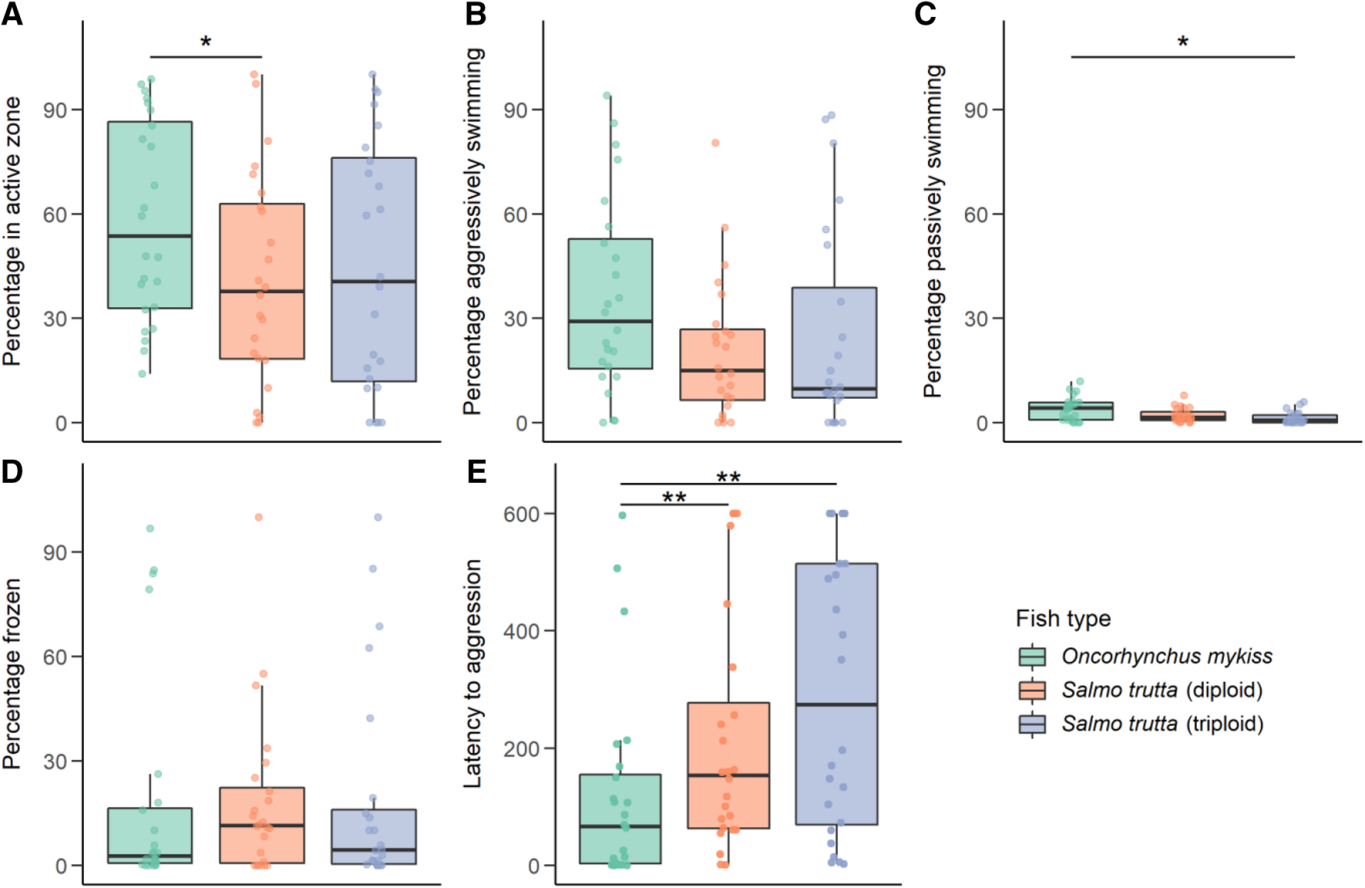
Boxplots showing the variables measured during the mirror test, with median and interquartile range plotted and overlayed with raw data points. Asterisks show significance values (* p < 0.05, ** p < 0.01, *** p < 0.001). (**A**) Percentage time spent in the “active zone” adjacent to the mirror, (**B**) percentage of the time in the active zone spent aggressively swimming against the mirror, (**C**) percentage of the time in the active zone spent passively swimming away from the mirror, (**D**) percentage of the time in the active zone spent being still, (**E**) latency to initiate aggressive swimming against the mirror.

Rainbow trout spent a significantly greater proportion of time in the active zone near the mirror than diploid brown trout (t = 2.40, df = 65, *p* < 0.05). Neither rainbow trout nor diploid brown trout differed significantly from triploids in proportion of time spent in the active zone (Fig. 4A). Fish types did not differ significantly in aggression or freeze behaviour, but triploid brown trout spent a smaller proportion of time swimming passively away from the mirror than rainbow trout (t = 2.48, df = 66, *p* < 0.05) and almost significantly more than the diploid brown trout (t = 2.26, df = 66, *p* = 0.068) (Fig. 4B–D). The order of the test was also significant, with the proportion time in the active zone spent aggressively swimming decreasing as the test took place later in the day (2.69, df = 65, *p* < 0.01), and the proportion time spent in freeze behaviour increasing with order in the day (t = 2.24, df = 65, *p* < 0.05). Rainbow trout were significantly faster to initiate aggressive swimming along the mirror than diploid brown trout (t = 3.17, df = 65, *p* < 0.01), and triploid brown trout (t = 3.16, df = 65, *p* < 0.01) with no difference between diploid and triploid brown trout (Fig. 4E). Heavier fish were also faster to initiate this aggressive behaviour (t = 2.40, df = 65, *p* < 0.05).

The results of this test show rainbow trout to be more aggressive across two measures compared with diploid brown trout, and across one measure compared with triploid brown trout.

### Feeding test

Feeding rate did not differ significantly by fish type or mass of fish. Only one variable was found to significantly improve the null model explaining feeding rate, the latency to initiate aggressive swimming in the mirror test, with the effect approaching significance (t = 1.76, df = 64, *p* = 0.08) (Fig. S1).

### Correlational analysis

#### Rainbow trout

The association between activity and boldness in rainbow trout was unclear, with both directions of correlation observed. The percentage time spent swimming in the open field test was negatively correlated with the percentage time spent outside the shelter (r = − 0.45, df = 22, *p* < 0.05), however, the inverse of the latency (1/latency) to approach the novel object was positively correlated with number of shelter crosses (r = 0.60, df = 22, *p* < 0.01). Boldness was negatively correlated with aggression along one measure, as shown by a negative correlation between percentage time spent in the inner rings in the novel object test and the percentage time in the active zone of the mirror test spent aggressively swimming (r = − 0.56, df = 22, *p* < 0.01). Activity was positively correlated with aggression along one measure: number of line crosses in the novel object test with the percentage time spent in the active zone of the mirror test (r = 0.48, df = 22, *p* < 0.05). No significant correlations were found across the behaviours with mass of the fish or number of bloodworms eaten. All correlations for rainbow trout are illustrated in Supplementary Fig. S2.

#### Diploid brown trout

Activity correlated with boldness in diploid brown trout, as shown by a single correlation between the 1/latency to approach the novel object and the number of shelter crosses (r = 0.61, df = 22, *p* < 0.01). There was also a correlation between activity and aggression in diploid brown trout, with the number of shelter crosses significantly correlating with 1/latency to begin aggressive swimming (r = 0.43, df = 22, *p* < 0.05). There were, however, no significant correlations between boldness and aggression variables, or for the number of bloodworms eaten with any of the behavioural measures. The mass of fish positively correlated with a boldness measure: percentage time spent in inner rings (r = 0.42, df = 22, *p* < 0.05), and an activity measure: number of line crosses in the novel object test (r = 0.45, df = 22, *p* < 0.05). All correlations for diploid brown trout are illustrated in Supplementary Fig. S3.

#### Triploid brown trout

Activity positively correlated with boldness in triploid brown trout, as shown by two measures: percentage time spent swimming in the open field test with percentage time spent outside the shelter (r = 0.59, df = 22, *p* < 0.01), and 1/latency to move in the open field test with the number of shelter crosses (r = 0.50, df = 22, *p* < 0.05). There is a positive correlation between activity and aggression, as shown by the association between the percentage time spent swimming in the open field test with 1/latency to begin aggressive swimming (r = 0.42, df = 22, *p* < 0.05), the number of shelter crosses with 1/latency to begin aggressive swimming (r = 0.53, df = 22, *p* < 0.01) and between the percentage time spent aggressively swimming in the active zone of the mirror test with the number of shelter crosses (r = 0.51, df = 22, *p* < 0.05), the number of line crosses in the novel object test (r = 0.44, df = 22, *p* < 0.05), and the percentage time spent swimming in the open field test (r = 0.54, df = 22, *p* < 0.01). There was however, a negative correlation between the percentage time spent in the active zone in the mirror test and the number of shelter crosses (r = − 0.46, df = 22, *p* < 0.05). Boldness also correlated with aggression as shown by a positive correlation between the percentage time spent being aggressive in the active zone of the mirror test and 1/latency to move in the open field test (r = 0.52, df = 22, *p* < 0.05), and with the percentage time spent outside the shelter (r = 0.41, df = 22, *p* < 0.05). Heavier triploid brown trout were more aggressive, as shown by a positive correlation between mass and 1/latency to begin aggressive swimming (r = 0.49, df = 22, *p* < 0.05). Fish with a higher feeding rate were also less active and less aggressive, as shown by negative correlations with the percentage time spent swimming in the open field test (r = − 0.43, df = 22, *p* < 0.05) and 1/latency to begin aggressive swimming (r = − 0.42, df = 22, *p* < 0.05). All correlations for triploid brown trout are illustrated in Supplementary Fig. S4.

### Group test

The best model for the group test included fish type and mean mass, but no interaction between fish type and type of test (disruption or novel object) (Fig. 2C). Rainbow trout had a significantly faster latency of the first fish to approach the central ring than diploid brown trout (t = 3.71, df = 14, *p* < 0.01), and triploid brown trout (t = 3.19, df = 14, *p* < 0.05) (Fig. 2C), with no significant difference between diploid brown trout and triploid brown trout. There was an almost significant effect of mean mass, with heavier groups with a faster latency (t = 1.86, df = 14, *p* = 0.08).

### Functional response trials

**Figure 5:**
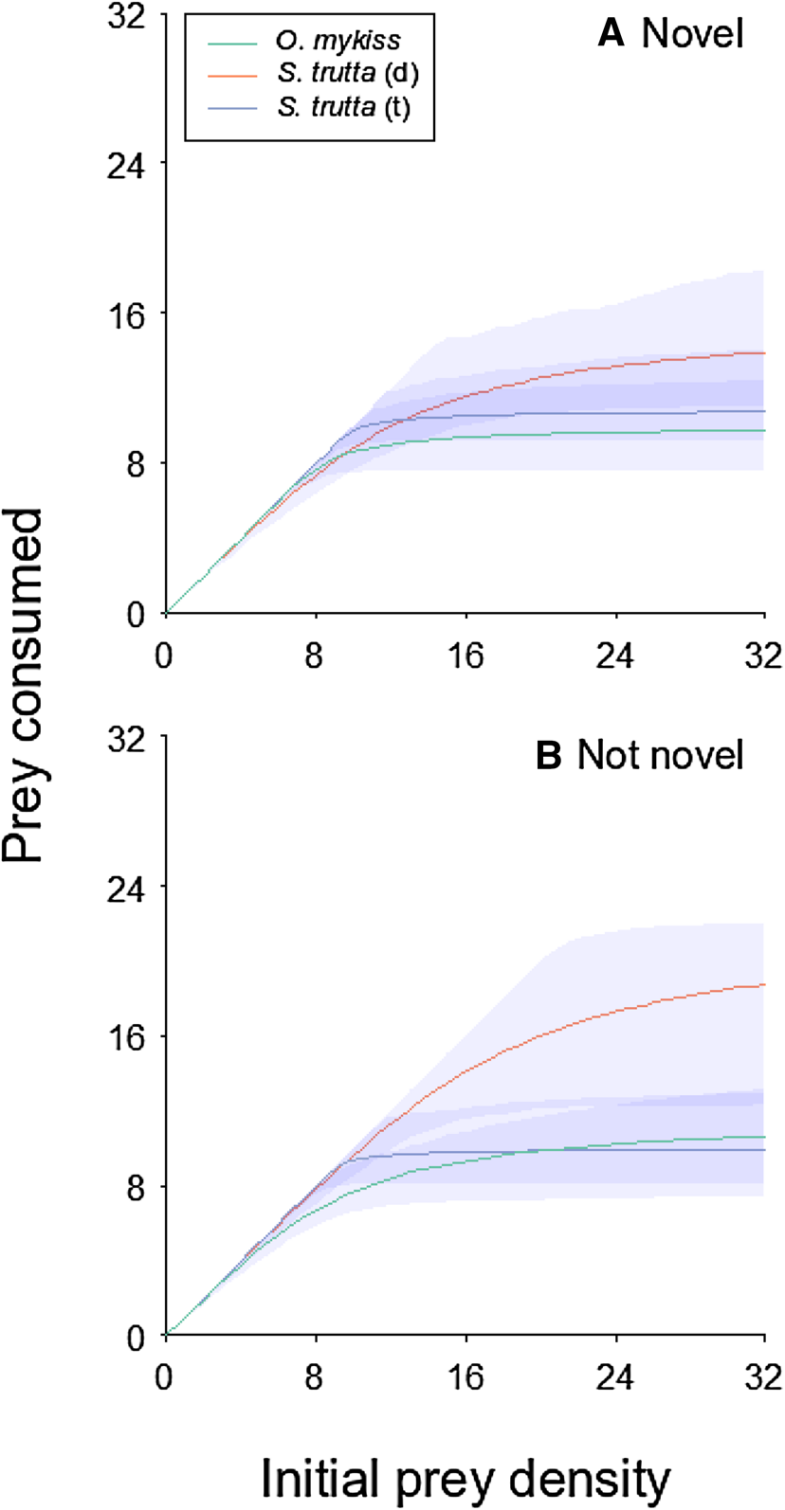
Functional Responses of diploid rainbow trout fry, diploid brown trout fry, and triploid brown trout fry under (**A**) novel prey conditions (i.e. fry had never fed on live chironomid prey previously) and (**B**) not novel conditions (i.e. fry had experienced live chironomid prey previously as part of the behaviour experiment). Clouds around lines represent bootstrapped 95% confidence intervals.

Prey survival in the predator-free controls was 100%, and therefore experimental deaths were attributed to predation. For all predators, Type II FRs were recorded, with significantly negative first-order terms in each instance (Table 1; Fig. 5). Under the novel prey treatment, triploid brown fry had the highest attack rates (triploid > diploid > rainbow), while diploid brown fry had the shortest handling times, and therefore highest maximum feeding rates (diploid > triploid > rainbow; Fig. 5a). For non-novel prey, triploid brown fry had the highest attack rates (triploid > diploid > rainbow), with diploid brown fry having the shortest handling times/highest maximum feeding rates (diploid > rainbow > triploid; Fig. 5b).

**Table 1:** First-order terms calculated from logistic regression to denote functional response type across all predator treatments.

Fish type	Novelty treatment	First-order term	Attack rate (*a*), *p* value	Handling time (*h*), *p* value	Maximum feeding rate (1/*h*, prey per 4 h)
Rainbow diploid	Novel	− 0.123***	13.995	0.100***	10.000
Brown diploid	Novel	− 0.118***	4.720***	0.063***	15.873
Brown triploid	Novel	− 0.122***	33.970***	0.092***	10.870
Rainbow diploid	Not novel	− 0.070***	4.223**	0.085***	11.765
Brown diploid	Not novel	− 0.106***	5.873**	0.045***	22.222
Brown triploid	Not novel	− 0.116***	40.915***	0.100***	10.000

The significantly negative first-order term values indicate Type II functional responses. Attack rate (*a*), handling time (*h*), maximum feeding rate (1/*h*) parameter estimates were derived using Rogers’ random predator equation (Eq. 1). *** p < 0.001, **p < 0.01. See also Fig. 5.

## Discussion

Several reports show increased levels of invasiveness and impact of brown trout compared with rainbow trout. Triploid individuals are also used to limit invasiveness and impact. We aimed to assess behavioural differences between rainbow trout and diploid and triploid brown trout to see if their behaviour may account for these reported differences. We show that rainbow trout are more active, shyer and more aggressive than diploid and triploid brown trout when tested individually, but in a group rainbow trout are bolder. Evidence for behavioural syndromes was weak, and the strongest evidence was found in triploid brown trout, with correlations between boldness, activity and aggression. The feeding rate results did not show any effect of trout type, but the comparative functional response experiments showed that diploid brown trout had the highest functional response, and that they increased their functional response to a greater degree when they had prior experience with the prey. Since functional responses allow us to examine feeding behaviour in more detail^16^, and have been related to impact across a range of taxa^56^ we have focused on these results more than the fixed density experiment.

Rainbow trout were more active than diploid and triploid brown trout, as shown by the increased percentage of time spent swimming and more line crosses in the open field test, and the greater number of line crosses in the disruption test. This pattern disappeared during the novel object test, and was likely due to the greater boldness of the diploid brown trout in the presence of the object, since the number of line crosses is directly related to approaches into the centre of the arena, and diploid brown trout were faster to approach the centre. The results of these tests indicate that the relative activity of rainbow and diploid brown trout do not generalise across contexts, with brown trout being more active in the presence of novel objects, but rainbow trout more active in a new environment. Furthermore, this may reflect different motivations towards activity, with brown trout moving to take advantage of novel phenomena, and rainbow trout moving in a new environment perhaps as a consequence of a greater stress response. Rainbow trout have been shown previously to exhibit higher stress responses to the same conditions compared with brown trout^57^. Short term cortisol treatment increases activity and does not decrease aggression in rainbow trout^45^, therefore the increased activity and aggression of the rainbow trout may suggest that the rainbow trout were more stressed than the brown trout during the experiment.

These results may also indicate that the brown trout were more affected by domestication than the rainbow trout, since it has been found previously that human protection can drive the coping style of low aggressiveness and low stress, with enhanced boldness, behavioural plasticity and cognitive abilities^58^. Alternatively the results may show that domestication has had contrasting effects within both lineages, since in rainbow trout aggressiveness has been shown to be associated with domestication^59^.

By being less active but more ready to respond to novel phenomena, domesticated brown trout may more effectively balance their energy requirements. This hypothesis makes sense when considering the feeding ecology of trout; both rainbow trout and brown trout fry typically capture food items as they drift past in the current. By remaining relatively still and only moving to approach novel items, brown trout may reduce their energy consumption while maximizing opportunities to feed. It has also been previously found that slower exploring brown trout grow faster since they expend less energy when foraging^52^. The behaviour of the rainbow trout was, however, not uniformly less adaptive than the diploid brown trout. Rainbow trout spent significantly more time under the shelter in the paired shelter/predation tests, suggesting that diploid brown trout may be more at risk of predation as a result of their greater boldness.

The presence of conspecifics had a greater impact on the behaviour of rainbow trout. For example, rainbow trout were more aggressive than both brown trout types, since they had a faster latency to initiate aggressive swimming in the mirror test. In the group test, rainbow trout were also bolder than both brown trout types, despite rainbow trout being shyer than both brown trout types when tested individually. However, a caveat of this test is that only the boldest fish was recorded in each case, rather than every fish in the group. These results may reflect an enhanced competitive drive influenced by the process of domestication, with aggression shown to be increased in hatchery but not wild rainbow trout fry previously^60^, or a reduction in the stress response of individuals leading to bolder behaviour. Rainbow trout are often released or escape in large numbers, so these results may mean that boldness may depend on the densities of introduced individuals. Evaluating how the density of fish affects the boldness of individuals and their vulnerability to predation is critical to assess the likelihood of survival of mass released and escaped exotic trout.

High functional responses of invaders compared to natives are excellent predictors of high ecological impact^12,15,61–63^. Based on observations in previous studies, there is some indication that brown trout are the more impactful invader compared with rainbow trout^22,28^, and our findings corroborate this, since the brown trout exhibit a higher functional response than the rainbow trout. Similarly, the triploid brown trout had a lower functional response than the diploids, suggesting that the triploidy treatment may lower the impact of brown trout. Although this comparative functional response experiment contradicted the results of the feeding test, which showed no differences between the three fish types, functional responses are better at assessing resource use^16^. The degree of impact was lessened when encountering a novel food source, suggesting that fish fed primarily on pellets may have a lesser impact when escaping into new environments with different food sources. Additionally, however, diploid brown trout showed a greater increase in functional response relative to the other fish types when exposed to a non-novel compared to a novel food source, suggesting that they may have a greater capacity for learning new food sources in the wild, contributing to their greater success and impact. The age-matching of trout in this study may have elevated the functional response of the slightly larger diploid brown trout, however, in the wild due to brown trout spawning earlier in the year, they likely would be considerably larger than rainbow trout of the same reproductive year. Therefore our comparison of impact is more conservative than what might be observed in the field, and our functional response results are entirely in line with known trout field impacts.

The importance of the behavioural variables in predicting impact is less clear, due to the contrasting results for boldness in the individual versus group conditions. Poorer management of energy reserves in escaped rainbow trout due to high aggression and activity may lead to excess mortality in the wild, leading to lower abundances and consequently low field impact. The increased activity may help to explain why in some studies rainbow trout is found to invade a wider range than brown trout^28^, despite having a smaller impact on natives.

Triploid brown trout were significantly less active and less bold than the diploid brown trout. This was evident from the significantly fewer line crosses during the open field trial and novel object test, more time spent in the outer ring in the open field test, fewer shelter crosses, and less time outside the shelter. More casual observations were also made of erratic behaviour by the triploid trout, with darting movements within the arena. There has been extensive comparison of physiology between diploid and triploid fishes, with significant differences found in stress and disease resistance^42^. For example, triploid Atlantic salmon have reduced gill surface area, potentially impacting ventilation, with triploids also having lower respiratory efficiency than diploids^40,64^, triploids also do not deal well with chronic stress^65^. A reduced ability to ventilate may have been a contributing factor to the differences in behaviour observed and, by reducing their activity, triploids may have been more effectively managing their own energy reserves. Alternatively, Atlantic salmon of differing ploidy showed no difference in stress responses^66^, therefore the different behaviour of the triploid brown trout may reflect cognitive differences compared to diploids. Previously no significant difference has been found between mixed-sex diploid and all-female triploid brown trout in terms of performance and survival when stocked, suggesting that in the wild any behavioural differences between the two varieties may have minimal consequences^41^.

A behavioural syndrome of activity with boldness and aggression in both brown trout varieties but not in rainbow trout, may indicate that correlations between these traits may be more advantageous in the brown trout lineage. Rainbow trout have been tested for a behavioural syndrome previously, but no evidence was found for a syndrome of dispersal, aggression and exploration^55^, confirming the lack of correlation of these traits in our study. The stronger correlation in triploid brown trout, as well as an association between boldness and aggression within this syndrome, may indicate that the triploid trout, due to their differences in physiology, present more extreme associations of correlated behaviours^36^. Alternatively, the difference between diploid and triploid brown trout may have been because we were unable to perform the correlations for the diploid brown trout using the open field test results.

A caveat of this analysis is that a wild analogue was not available for the species studied. Since all of the fish used in the experiment were domesticated strains it is not possible to disentangle the species and ploidy-level differences in behaviour from those due to the domestication process. Furthermore, only fish from a single farm were used in the analysis, so it is not possible to determine whether the effects observed are generalizable to other farms. However, farmed varieties of trout are relatively homogenous genetically due to the large degree of interchange between farms over many years^67,68^, although it is possible that the two species of trout had slightly different degrees of domestication. Additionally, many of the successful invasions of both species worldwide occurred several decades ago, and would have involved strains closer in phenotype to the wild source strains. However, the effects of domestication typically take effect within a handful of generations^37^, and it is highly probable that the strains of rainbow and brown trout used in this study were domesticated over many generations, making any differences in the degree of domestication relatively small.

Additionally, many trout are released at an age much older than the fry stage when they are even more habituated to the farm environment and so may be less likely to adapt in the wild. The changes to behaviour over time may also differ between the three trout types, meaning that the trends observed here in fry may not be the same for older fish. Mass escapes of all age groups do, however, occur, as evidenced by a mass escape of over 300,000 rainbow trout into the River Strule, Northern Ireland from a fish farm comprising all age classes of fish^69^. Finally, it is difficult to determine whether the behavioural differences here would last over multiple generations of released individuals, since it is challenging to determine the heritability of behavioural traits. However, with continuous release of stocked varieties over time, the assessments are still relevant with respect to impact, since impact may take place in the absence of the establishment of wild populations of these stocked fish.

Despite these caveats, our findings suggest that brown trout may be more impactful, due to a higher functional response, and that the species’ combination of behavioural traits may make it more likely to survive in the wild. This may explain why in certain scenarios, brown trout has been shown to be the more impactful invader compared with rainbow trout^28^, since high functional response and high population densities predicts high impact^56^. The findings that domesticated rainbow trout are shyer and have lower functional responses than brown trout may help to explain the lack of success and ecological impact of rainbow trout, especially in areas like the British Isles where the native wild brown trout is likely to be far better adapted to the local conditions, and so may limit invasion potential of the rainbow trout. Brown trout is typically seen as a safer option for stocking in Europe compared with rainbow trout since it is native to the area, however, with a higher functional response and greater success as an invader elsewhere, regular stocking may in fact be more detrimental to the environment compared with rainbow trout stocking, something which fisheries managers may wish to take into account. We present evidence, however, that triploid brown trout may alleviate some of these impacts. Alternatively, the greater activity and aggression of rainbow trout may mean that they are more disruptive through other means, such as disturbing native fish and attracting predators.

Our results demonstrate the value in combining behaviour tests with comparative functional response tests to evaluate the risks posed by invasive species, and to explore the reasons for differences in survival and impact. Furthermore, we show that these tests can even highlight significant differences between two highly domesticated species with a similar invasion history. Further research measuring differences in cortisol levels of both domesticated species under these experimental conditions would be useful, as well as a comparison between wild-type rainbow trout and brown trout with their respective domesticated relatives. Inclusion of triploid rainbow trout into the comparison would be of interest (and was not possible for this study) since they are also released into the wild. Changes in temperature and other environmental variables may also modify the behaviour of these species affecting their impact^70^, therefore performing these experiments under different temperature treatments would also be advantageous. This additional work would help to determine the relative importance of the processes of domestication and natural selection in shaping the differences between these species, and in combination with field-based studies would further help to elucidate the reasons behind the differences in the relative success and impacts of rainbow trout and brown trout as invasive species.

## Methods

### Collection and maintenance

Diploid rainbow trout fry (mean mass ± SE: 0.418 ± 0.0325 g), diploid brown trout fry (0.602 ± 0.0563 g), and triploid brown trout fry (0.470 ± 0.0311 g) were acquired from Movanagher Fish Farm, Northern Ireland. Each fish type was acquired in a single batch of 100 fish approximately 8 weeks after the official hatch date of each type, between February to April 2019, and held in the laboratory for 3–5 days prior to any behavioural testing. Four fish were tested on each day up to 12 days after the testing began, with 6 days of testing in total per fish type (giving 6 × 4 = 24 individuals tested per fish type). We chose to age-match rather than size-match to test the fry at the same developmental stage with the same experience, however, we also used mass of fish in our statistical modelling of all behaviour variables to control for any size-effects. This method in which fish size is not controlled has been used in other studies comparing personality between species^71^. Fry were moved to the Queen’s University Belfast Medical Biology Centre, where they were held in a 12 °C laboratory with a 12:12 light regime, with complete water changes every two days. Fry were housed in two holding tanks (39.5 × 25 × 27 cm) with approximately 50 fish in each tank for the duration of the experiment and were fed ad libitum twice daily with INICIO Plus 0.5 mm food pellets from the fish farm. Morning feeding took place after four fish had been selected for the day’s experiments so that fish used in experiments were starved for approximately 15 h from the previous day.

### Ethical statement

Fish were kept in densities lower than those in the fish farm from which they were acquired (fish farm: approx. 41,269–61,904 fish per m^3^, our tanks: 1,872 fish per m^3^) and, during transfer between the holding tanks and the experimental arenas, the time spent out of the water was minimised to less than 10 s. This work did not fall under the definition of regulated procedures as per the UK Animals (Scientific Procedures) Act 1986, however, all experiments adhered to UK regulations and institutional ethical approval was granted by the Queen’s University Belfast, School of Biological Sciences Research Ethics Committee, and experiments were also conducted following recommendations in ARRIVE guidelines and all other relevant guidelines and recommendations. After the end of the study, fish were returned to Movanagher fish farm and kept isolated from the other fish on the farm for a week to prevent possible transfer of disease.

### Behavioural trials

Each trial involved four fish being chosen at random from a holding tank, with each placed in 2L of dechlorinated tap water (previously oxygenated overnight and until usage in trials) in one of four white buckets (henceforth “arenas”), surrounded by cardboard screens to ensure shading from direct light. All four fish were videoed from above simultaneously during each trial. At the bottom of each arena two concentric circles marked out three regions: the outer ring, inner ring and centre (Supplementary Fig. S5). After introduction to the arenas on each day, fry were given a 15-min adaptation period. This adaptation period was filmed and served as an open field test within the study. After this open field test, one of our three additional behaviour tests (i.e. disruption/novel object, shelter/predation or mirror tests) began, with the order of the trials on each day alternating.

Each part of each behaviour test consisted of a 15-min recorded period. After fish were disturbed by transitions between each test, they were left to recover for 15 min before beginning the next test. The open field test and mirror test consisted of only one 15-min part, but the novel object and shelter tests were further subdivided into two components, each filmed for 15 min. After all behaviour tests were completed all four fish were subjected to a feeding trial, then weighed. Finally, each fish was placed into one of the original arenas to form a group of four conspecifics, with the group then filmed for an open field test, and the two components of the novel object test. See Supplementary Fig. S6 for a full flow chart of the behaviour tests. All response variables to be used in statistical analyses obtained from these experiments are shown in Table 2. After the behaviour trials and feeding tests had taken place, individuals involved in these trials were kept in a separate tank for 2–5 days prior to the functional response trials (see below).

#### Open field test

Fish were released into the arena at the start of the day and their reaction to the new environment was recorded. For each video, the percentage time spent swimming versus resting and the total number of line crosses between the locations in the arena (whether in the outer ring, inner ring or centre) where recorded as measures of activity. The latency to start swimming and the percentage time in each location were also recorded as measures of boldness. Where fish stayed still for the entirety of the video, latency was recorded as 600 s (the total length of the video).

#### Disturbance/novel object test

This paired test consisted of two 15-min components. In the first component (disturbance test), a blue plastic airline splitter was quickly tapped on the surface of the water for each arena; this was to mimic the disturbance to the fish of the novel object being introduced into the arena. In the second component of the test (novel object test), the same object was lowered into the central circle of the arena. For both tests, activity was measured as the total number of line crosses between arena locations. The latency to enter the central ring and the percentage time spent in each location of the arenas were recorded as measures of boldness. In cases where the fish did not enter the central ring during the trial, the latency was recorded as 600 s (the length of the video).

#### Shelter/predation test

Cardboard screens around the arenas were removed prior to the test. The test proceeded with two 15-min components. For both parts a cardboard sheet was placed over 50% of the area of each arena (Supplementary Fig. S5C). In the first part (shelter test), fry were filmed in the presence of the shelter and with no other disturbance. In the second part (predation test), a person was present throughout the test and waved their hands above the arenas every 30 s for approximately 5 s to simulate a predation threat. Fish were recorded as either under or outside the shelter. The number of transitions between inside and outside the shelter were recorded as a measure of activity, and percentage time outside the shelter recorded as a measure of boldness.

#### Mirror test

A square mirrored tile (15 × 15 cm) was lowered and fixed against the side of each arena with adhesive putty (Supplementary Fig. S5D). During video analysis, an active zone adjacent to the mirror was defined as between the mirror and the line outlining the outer ring. The percentage time spent within or outside the active zone was recorded. In addition, the percentage of the time within the active zone spent doing the following three behaviours was also calculated: active behaviour (swimming while oriented to the mirror), passive behaviour (swimming away from the mirror), and freeze behaviour (lying still). The latency to start swimming alongside the mirror was also recorded as a separate measure of aggression.

#### Feeding test

Fry were moved from their arenas, and placed in transparent food containers (14.5 × 9.5 × 4 cm dimensions and volume 400 ml) with 10 bloodworms (*Chironomidae* spp.). Trials ran for four hours, with the number of alive prey remaining counted afterwards. Fry were then weighed using electronic scales.

**Table 2:** List of response variables from each of the behaviour tests, and the behavioural descriptors for each variable.

Test	Response variable	Behaviour
Open Field test	Latency to swim	Boldness
	Total line crosses	Activity
	Percentage time swimming	Activity
	Percentage time in outer ring	Boldness
	Percentage time in inner ring	Boldness
	Percentage time in centre	Boldness
Disturbance and novel object test	Latency to approach	Boldness
	Total line crosses	Activity
	Percentage time in outer ring	Boldness
	Percentage time in inner ring	Boldness
	Percentage time in centre	Boldness
Shelter and predation test	Percentage outside shelter	Boldness
	Shelter crosses	Activity
Mirror	Latency to begin swimming along mirror	Aggression
	Percentage in active zone	Aggression
	Percentage time swimming in active zone	Aggression
	Percentage time passive in active zone	Aggression
	Percentage time frozen in active zone	Aggression
Group test	Latency of first fish to approach	Boldness

#### Group test

The four fish involved in the simultaneous trials each day were introduced into a single arena. After all four fish had been introduced, the cardboard screens were placed around the arena and the fish were left to acclimatise for 15 min. The disturbance test and novel object test were then conducted as for the individual tests, this time using a small, purple, plastic figurine as the novel object. The latency of the fastest fish to approach the central circle was then recorded.

#### Video analysis

The final 5 min of each video were removed to give 10 min videos for analyses, using Shotcut video editing software. This was performed to make analyses of all videos feasible in the time available and because preliminary viewings did not show dramatic changes in behaviour in the final five minutes. The open field test was cut to start immediately after the person setting up the arenas left the room. All other tests were trimmed to start 10 s after the final disturbance to each of the fish. BORIS v.7.9.7 was used to perform all behaviour analyses^72^. The position of each fish was determined by the location of the fish’s head. Data were compiled using R v.3.6.2^73^, in RStudio v.1.2.5033^74^.

### Functional response trials

The functional response of all three trout types to bloodworms as prey were measured, with two levels of experience also tested. Fry that were naïve to bloodworms (i.e. had not been used in the behavioural trials) were used to measure the functional response to “novel” prey. Fry with prior experience of bloodworms from the behavioural trials were used to measure the functional response to “not novel” prey. Both groups of fry were kept in separate holding tanks and starved overnight (approximately 15 h). Individual fry were placed in arenas (14.5 × 9.5 × 4 cm dimensions and volume 400 ml) which were made visually uniform by having masking tape wrapped around the outside. Transparent lids were placed on top of these containers to prevent escape of fish and green plastic sheets were placed around the arenas to prevent additional visual stimuli. Six densities of prey were used (1, 2, 4, 8, 16 and 32: n = 3 per prey density per trout type per experience level) and fry were added to these arenas and allowed to feed for four hours (similar design to Alexander et al. 2014), with the number of alive prey counted afterwards. Controls were performed (n = 3 per prey density) with the same experimental conditions but in the absence of fry, to quantify prey mortality for any other reasons.

### Statistical analyses

#### Individual tests

As preliminary modelling revealed heteroscedasticity in some of the residuals for the models, for each of the response variables from the behaviour tests, model selection was performed following the protocol outlined in Zuur et al. (2008)^75^. All models were fitted using the lme function in the nlme package^76^ in R^73^. All data compilation was performed using the dplyr package^77^ and plots generated using ggplot2^78^.

Mixed effects models were fitted using two explanatory variables: type of fish (i.e. diploid rainbow, diploid brown, or triploid brown) and mass of each individual fish. For the disruption/novel object and shelter/predation tests the component of the test was also included as a factor (i.e. the first versus the second 15-min segment of the test) with an interaction term between the test component and the type of fish. Since the order of the paired tests and the mirror test varied (Fig. S6), the order of test was also included as a numerical variable (i.e. whether the test was 1st, 2nd or 3rd on that day) to account for any time effects for these tests. All models contained a random factor of Arena, while the paired disruption/novel object and shelter/predation tests also contained individual as a random factor nested within Arena. Since heterogeneity was observed across fish type for several response variables, a variance structure varying by fish type was also included and a likelihood ratio test used to determine its significance. In total, 19 response variables were modelled in this way (Table 2). All proportion data were arcsine square-root transformed and number of line crosses log_10_ transformed prior to modelling for better distribution of residuals in resulting models. Latency (+ 1) data were also log_10_ transformed, except for the novel object test where residuals for the latency models were more normally distributed using a gaussian distribution. The dredge function^79^ was used on all global models (after variance structure was selected) to determine the most parsimonious in all cases, with the top model reported by dredge being determined the most parsimonious model. Contrasts across different factor levels among any interactions included in the final models were calculated using the emmeans package^80^ , using the containment method for degrees of freedom estimation.

#### Feeding test

The number of bloodworms eaten by the fish was modelled with respect to the type of fish, fish mass and the response variables from the previous behaviour tests for which a significant difference was found between the fish types. Model selection was carried out using the Zuur et al. (2008) protocol as above.

#### Correlational analysis

For each fish type, Pearson’s correlations were performed for a selection of variables describing activity, boldness and aggression. Only combinations of different behaviour types were considered in the analysis, i.e. bold traits with active traits, as well as correlations between these variables and fish mass and number of bloodworms eaten. Additionally, only correlations which involved combinations of different tests were considered, since some variables are not independent within each test (i.e. latency to approach object and number of line crosses in the novel object test). The open field test results were also not included for diploid brown trout, because due to complications in the beginning of the study a subset of the fish used for the open field test were not also used for the other tests (although all fish experienced identical experimental conditions).

#### Group test

The latency of the first fish to approach the central circle was modelled with respect to fish type, whether the disturbance or novel object test, and the mean mass of the fish in the test. An interaction term between fish type and type of test was also included in the most complex model, and date tested (and therefore group ID) was included as a random effect. Model selection was carried out using the protocol as above.

#### Functional response trials

Functional Responses (FR) were modelled using the ‘frair’ package^81^. The FR curve types (Type I, II or III^16^) were derived through logistic regression of the proportion of prey consumed as a function of prey density offered^82^. Significantly negative first-order terms indicate hyperbolic Type II curves, whereas significantly positive first-order terms followed by significantly negative second-order terms indicate sigmoidal Type III curves. Rogers’ random predator equation was used to model FRs since prey were not replaced as they were consumed^83^:

1 $${N}_{e}={N}_{0}(1-\mathrm{exp}(a\left({N}_{e}h-T\right)))$$

where *N*_*e*_ is the number of prey eaten, *N*_0_ is the initial density of prey, *a* is the attack constant, *h* is the handling time and *T* is the total experimental period (i.e. four hours). Maximum feeding rates (1/*h*) were calculated under each treatment group. The *Lambert W* function was used to solve the random-predator equation^84^. We generated 95% confidence intervals around FR curves using non-parametric bootstraps (*n* = 2000).

#### Inter-rater repeatability test

JWED analysed all mirror test videos, JWED and CLOM analysed all open field test videos and student assistants SD, and CAN with CLOM analysed all remaining tests. For those tests in which more than one person was involved in the analysis, 10% of videos were analysed by all observers. A statistical model including arena, observer and fish type as explanatory variables was run for each variable. In each case there was no significant effect of observer, except for one: the number of line crosses in the paired disturbance/novel object test. Because there was a significant effect of observer in this case, the modelling for this variable was repeated including observer as an explanatory variable, but there was no effect on the significance of the other variables in the model.

## Supplementary Information

Supplementary Information 1.

Supplementary Information 2.

Supplementary Information 3.

Supplementary Information 4.

Supplementary Information 5.

Supplementary Information 6.

Supplementary Information 7.

Supplementary Information 8.

Supplementary Information 9.

## Data Availability

All data generated or analysed during this study are included in this published article (and its Supplementary Information files).
