# Supplementary material for: Behavioural traits of rainbow trout and brown trout may help explain their differing invasion success and impacts
Source: Sci Rep. 2022 Feb 2;12:1757. doi: 10.1038/s41598-022-05484-5 (PMC8810905; doi:10.1038/s41598-022-05484-5)
Supplement: Supplementary file 1 — Supplementary Information 1. [file 41598_2022_5484_MOESM1_ESM.pdf]

Behavioural traits of rainbow trout and brown trout may help explain their differing invasion success and impacts

Ciara L. O. McGlade[1*], James W. E. Dickey[1,2,3], Richard Kennedy[4], Shannon Donnelly[1], Clare-Ann Nelson[1], Jaimie T. A. Dick[1] & Gareth Arnott[1]

[1] *Institute for Global Food Security, School of Biological Sciences, Queen's University Belfast, 19 Chlorine Gardens, Belfast,* Northern Ireland, *BT9 5DL, UK*

[2] *Leibniz-Institute of Freshwater Ecology and Inland Fisheries (IGB), 12587 Berlin, Germany*

[3] *Institute of Biology, Freie Universität Berlin, 14195 Berlin, Germany*

[4] *AFBI Aquatics Group, River Bush Salmon Station, Church Street, Bushmills, Northern Ireland, BT57 8QJ*

[*] *Corresponding author*

Supplementary figures

Figure S1

Boxplots showing the number of bloodworms eaten during the feeding test by each trout type, with median and interquartile range plotted and overlayed with raw data points.

Figure S2

Pearson's R coefficient for Rainbow trout for each of the correlations performed (significant correlations have colours, NA = no correlation performed due to overlapping test or behaviour variable (i.e. both boldness variables, or both from novel object test), white = correlation performed but not significant, red= -ve correlation, blue = +ve correlation). A: "activity" variables given along the top axis, with the relevant correlations listed down the side (i.e. boldness and aggression variables), B: "boldness" variables following the same format (against activity and aggression variables), and C: the correlations of all of the behavior variables against the fish mass and feeding rate.

Figure S3

Pearson's R coefficient for diploid brown trout for each of the correlations performed (significant correlations have colours, NA = no correlation performed due to overlapping test or behaviour variable (i.e. both boldness variables, or both from novel object test), white = correlation performed but not significant, red= -ve correlation, blue = +ve correlation). A: "activity" variables given along the top axis, with the relevant

correlations listed down the side (i.e. boldness and aggression variables), B: “boldness” variables following the same format (against activity and aggression variables), and C: the correlations of all of the behavior variables against the fish mass and feeding rate.

Figure S4

Pearson’s R coefficient for triploid brown trout for each of the correlations performed (significant correlations have colours, NA = no correlation performed due to overlapping test or behaviour variable (i.e. both boldness variables, or both from novel object test), white = correlation performed but not significant, red= -ve correlation, blue = +ve correlation). A: “activity” variables given along the top axis, with the relevant correlations listed down the side (i.e. boldness and aggression variables), B: “boldness” variables following the same format (against activity and aggression variables), and C: the correlations of all of the behavior variables against the fish mass and feeding rate.

Figure S5

The experimental set-up for each of the behaviour tests. A: Open Field Test, B: Disturbance/Novel Object tests, C: Shelter/Predation tests, D: Mirror test, E: Group Disturbance/Novel object tests.

Figure S6

Flowchart illustrating the order of the individual and group tests. Functional response experiments were also performed separately on fish not involved in these experiments.

Figure S1

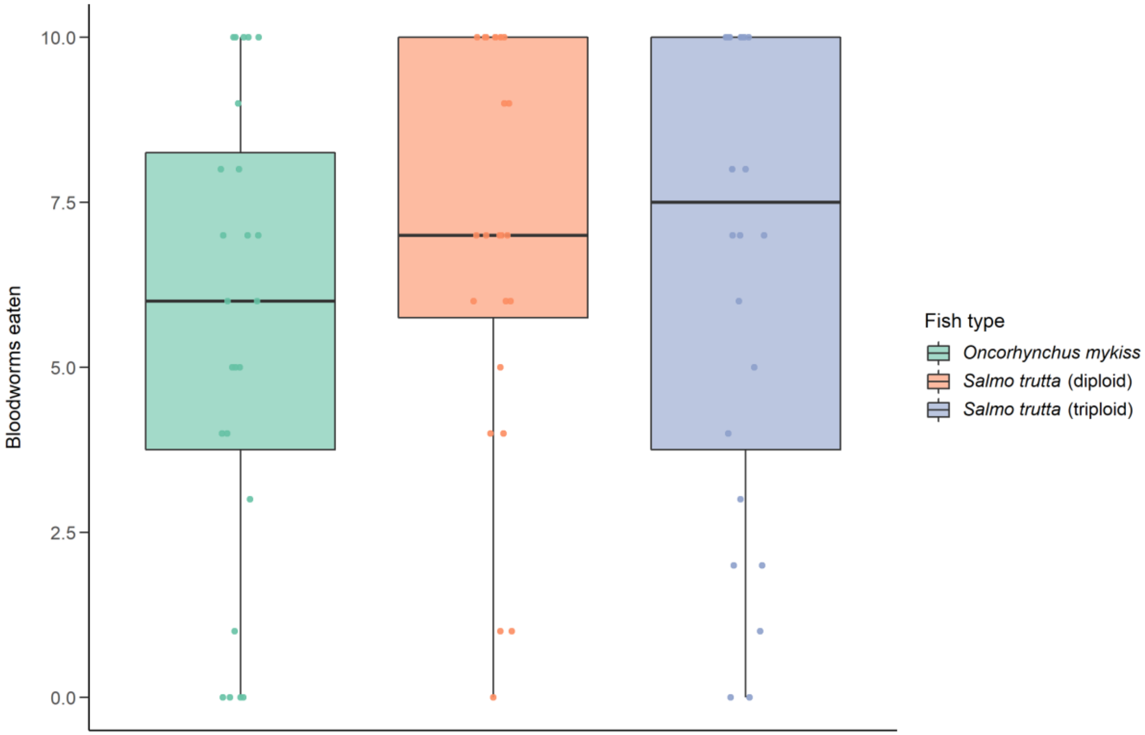
10.0
7.5
5.0
2.5
0.0
Bloodworms eaten
Fish type
Oncorhynchus mykiss
Salmo trutta (diploid)
Salmo trutta (triploid)

Figure S2

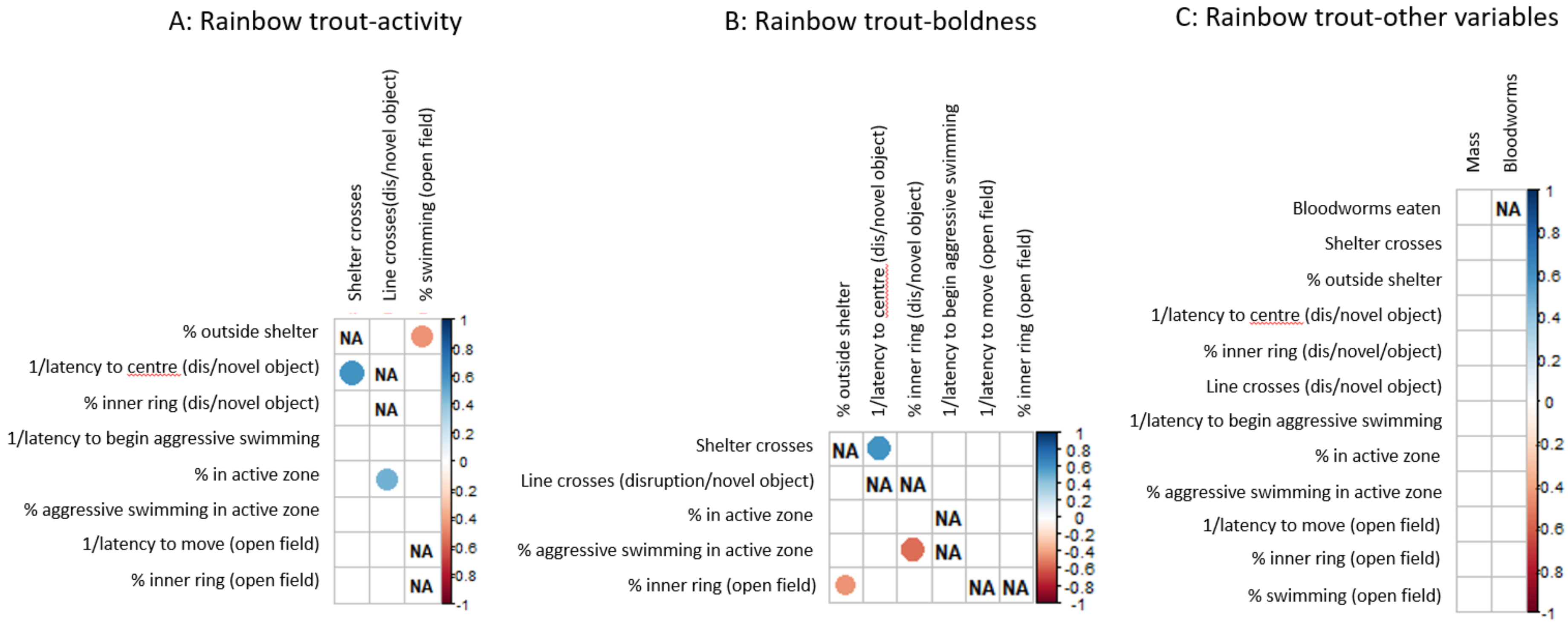
A: Rainbow trout-activity
Shelter crosses
Line crosses(dis/novel object)
% swimming (open field)
% outside shelter
1/latency to centre (dis/novel object)
% inner ring (dis/novel object)
1/latency to begin aggressive swimming
% in active zone
% aggressive swimming in active zone
1/latency to move (open field)
% inner ring (open field)
NA
B: Rainbow trout-boldness
% outside shelter
1/latency to centre (dis/novel object)
% inner ring (dis/novel object)
1/latency to begin aggressive swimming
1/latency to move (open field)
% inner ring (open field)
Shelter crosses
Line crosses (disruption/novel object)
% in active zone
% aggressive swimming in active zone
% inner ring (open field)
1
0.8
0.6
0.4
0.2
0
-0.2
-0.4
-0.6
-0.8
-1
C: Rainbow trout-other variables
Mass
Bloodworms
Bloodworms eaten
Shelter crosses
% outside shelter
1/latency to centre (dis/novel object)
% inner ring (dis/novel/object)
Line crosses (dis/novel object)
1/latency to begin aggressive swimming
% in active zone
% aggressive swimming in active zone
1/latency to move (open field)
% inner ring (open field)
% swimming (open field)

Figure S3

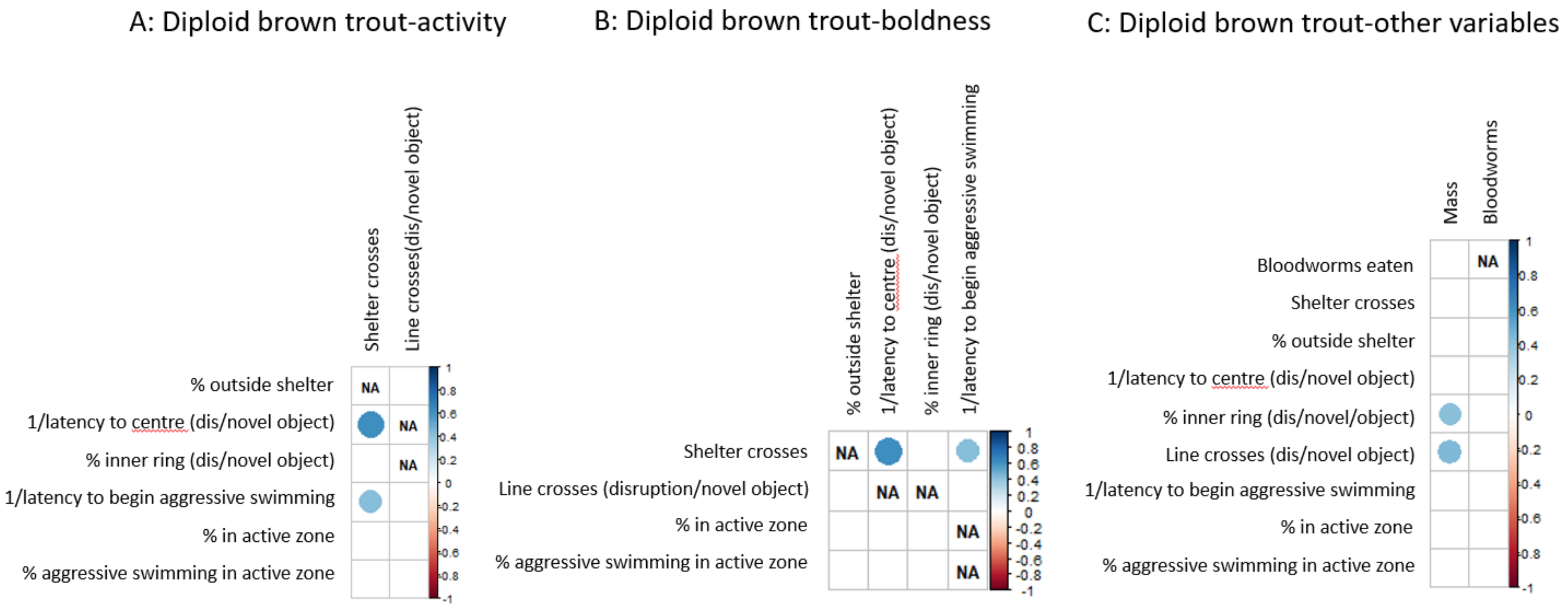

A: Diploid brown trout-activity
Shelter crosses
Line crosses(dis/novel object)
% outside shelter
1/latency to centre (dis/novel object)
% inner ring (dis/novel object)
1/latency to begin aggressive swimming
% in active zone
% aggressive swimming in active zone
NA
B: Diploid brown trout-boldness
% outside shelter
1/latency to centre (dis/novel object)
% inner ring (dis/novel object)
1/latency to begin aggressive swimming
Shelter crosses
Line crosses (disruption/novel object)
% in active zone
% aggressive swimming in active zone
C: Diploid brown trout-other variables
Mass
Bloodworms
Bloodworms eaten
Shelter crosses
% outside shelter
1/latency to centre (dis/novel object)
% inner ring (dis/novel/object)
Line crosses (dis/novel object)
1/latency to begin aggressive swimming
% in active zone
% aggressive swimming in active zone

Figure S4

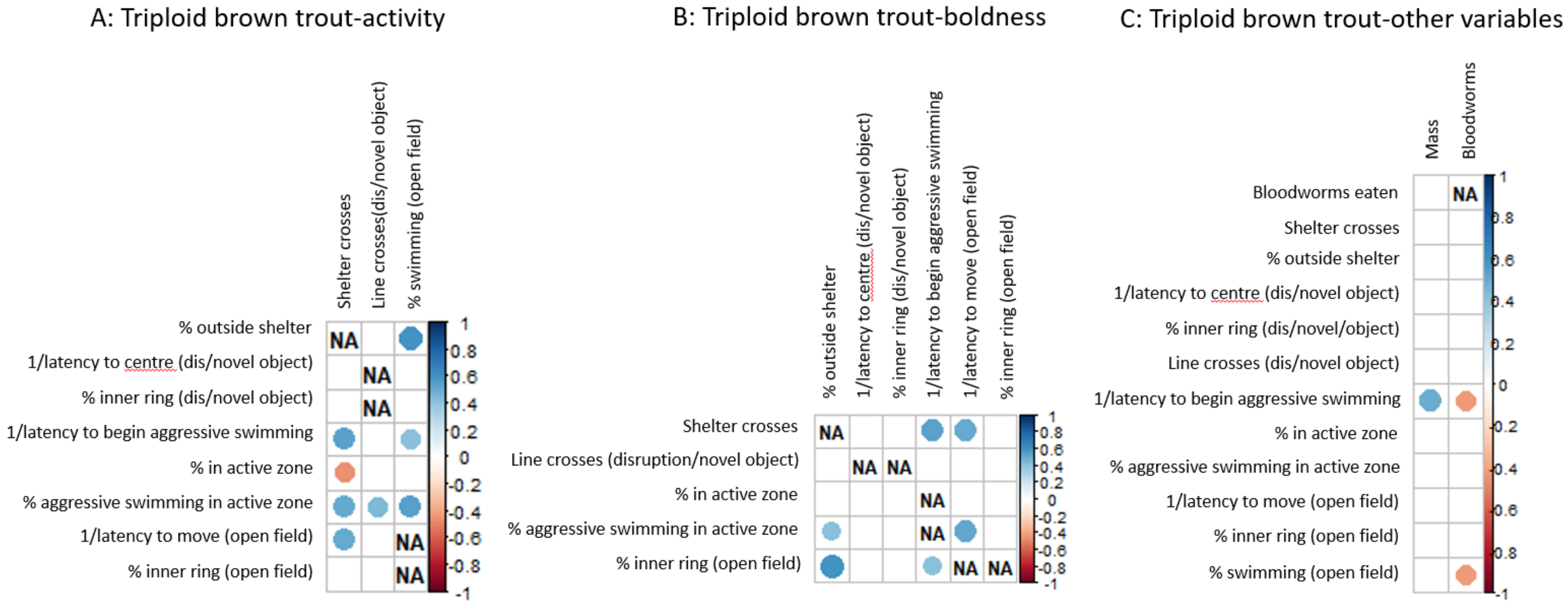
A: Triploid brown trout-activity
Shelter crosses
Line crosses(dis/novel object)
% swimming (open field)
% outside shelter
1/latency to centre (dis/novel object)
% inner ring (dis/novel object)
1/latency to begin aggressive swimming
% in active zone
% aggressive swimming in active zone
1/latency to move (open field)
% inner ring (open field)
NA
B: Triploid brown trout-boldness
% outside shelter
1/latency to centre (dis/novel object)
% inner ring (dis/novel object)
1/latency to begin aggressive swimming
1/latency to move (open field)
% inner ring (open field)
Shelter crosses
Line crosses (disruption/novel object)
% in active zone
% aggressive swimming in active zone
% inner ring (open field)
C: Triploid brown trout-other variables
Mass
Bloodworms
Bloodworms eaten
Shelter crosses
% outside shelter
1/latency to centre (dis/novel object)
% inner ring (dis/novel/object)
Line crosses (dis/novel object)
1/latency to begin aggressive swimming
% in active zone
% aggressive swimming in active zone
1/latency to move (open field)
% inner ring (open field)
% swimming (open field)

Figure S5

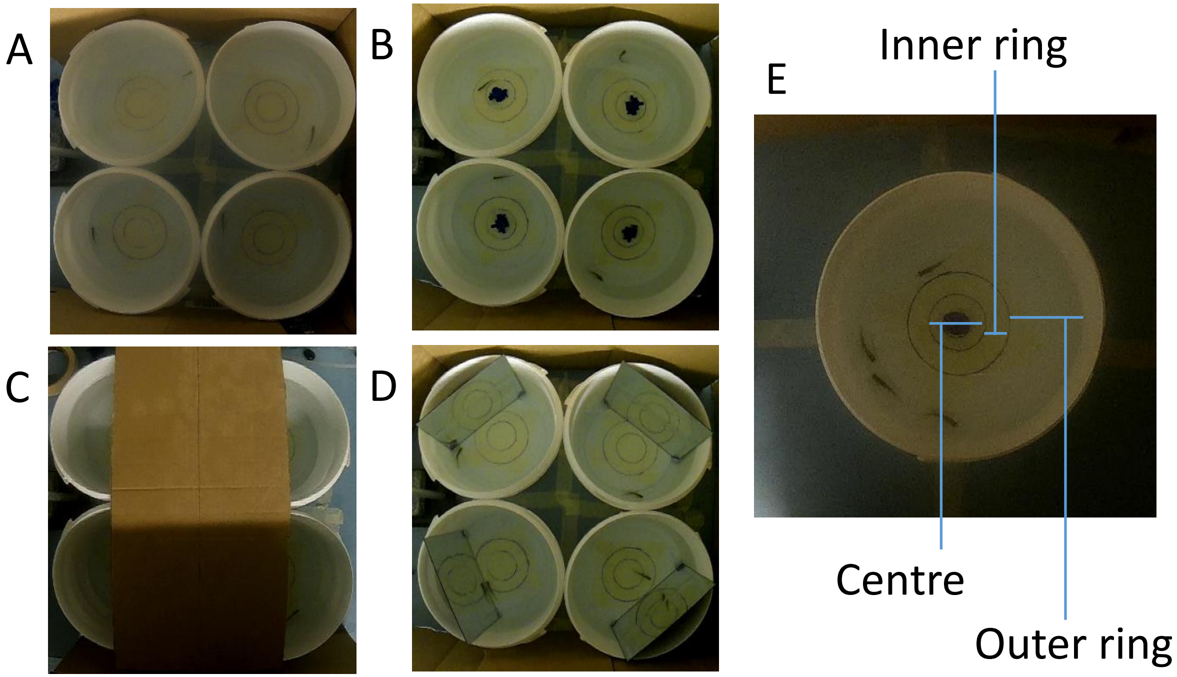

Figure S6

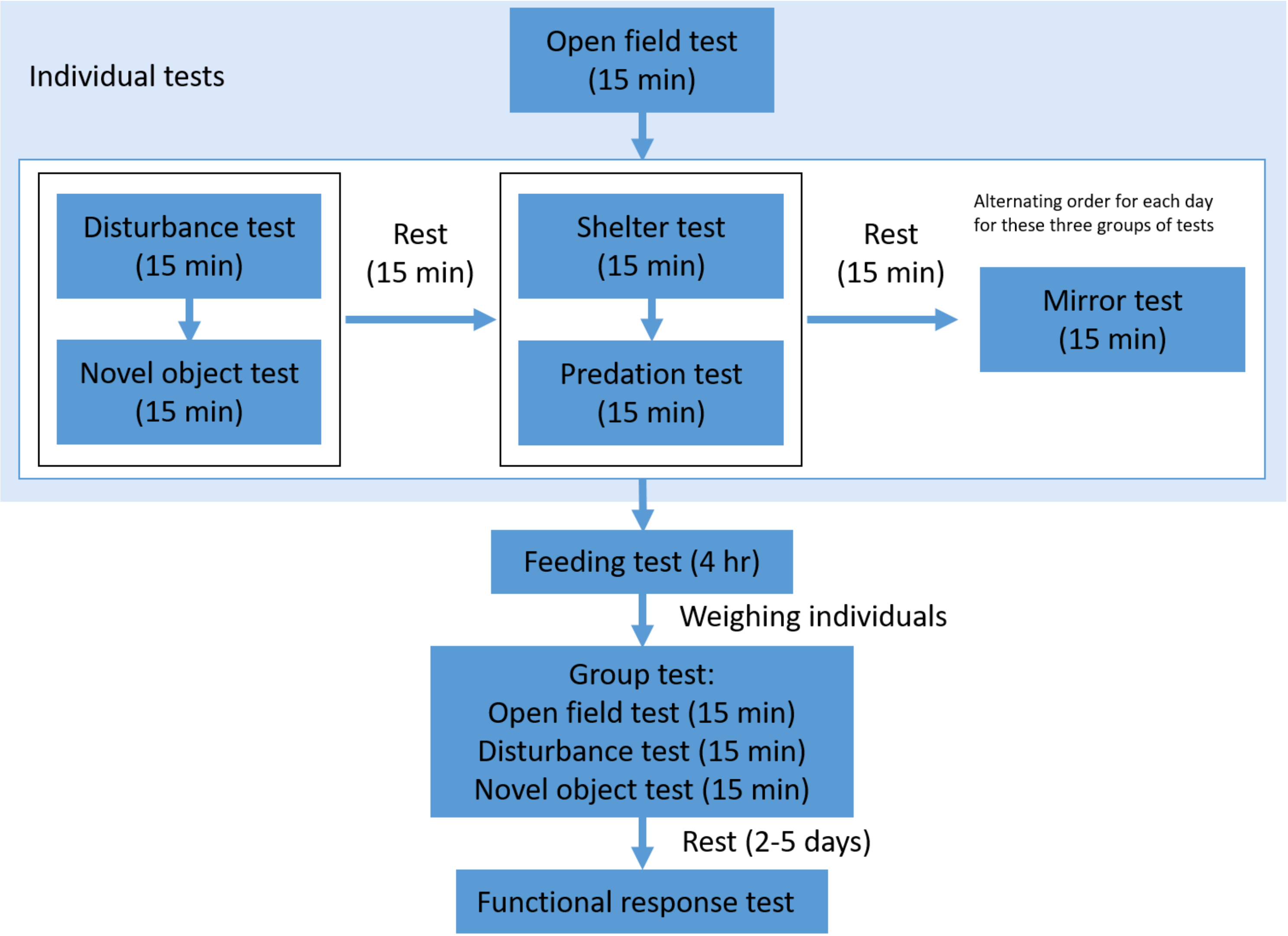

Individual tests
Open field test
(15 min)
Disturbance test
(15 min)
Novel object test
(15 min)
Rest
(15 min)
Shelter test
(15 min)
Predation test
(15 min)
Rest
(15 min)
Alternating order for each day
for these three groups of tests
Mirror test
(15 min)
Feeding test (4 hr)
Weighing individuals
Group test:
Open field test (15 min)
Disturbance test (15 min)
Novel object test (15 min)
Rest (2-5 days)
Functional response test
